# The impact of mitral valve surgery on ventricular arrhythmias in patients with Barlow’s disease: preliminary results of a prospective study

**DOI:** 10.1093/icvts/ivad073

**Published:** 2023-05-11

**Authors:** Guido Ascione, Nicolò Azzola Guicciardi, Roberto Lorusso, Antonio Boccellino, Elisabetta Lapenna, Benedetto Del Forno, Davide Carino, Arturo Bisogno, Anna Palmisano, Giuseppe D’Angelo, Paolo Della Bella, Antonio Esposito, Eustachio Agricola, Ottavio Alfieri, Alessandro Castiglioni, Francesco Maisano, Pasquale Vergara, Michele De Bonis

**Affiliations:** Department of Cardiac Surgery, IRCCS San Raffaele Hospital, Vita-Salute San Raffaele University, Milan, Italy; Department of Cardiac Surgery, IRCCS San Raffaele Hospital, Vita-Salute San Raffaele University, Milan, Italy; Cardio-Thoracic Surgery Department, Maastricht University Medical Centre, Cardiovascular Research Institute Maastricht, Maastricht, Netherlands; Echocardiography Laboratory, IRCCS San Raffaele Hospital, Vita-Salute San Raffaele University, Milan, Italy; Department of Cardiac Surgery, IRCCS San Raffaele Hospital, Vita-Salute San Raffaele University, Milan, Italy; Department of Cardiac Surgery, IRCCS San Raffaele Hospital, Vita-Salute San Raffaele University, Milan, Italy; Department of Cardiac Surgery, IRCCS San Raffaele Hospital, Vita-Salute San Raffaele University, Milan, Italy; Department of Cardiac Surgery, IRCCS San Raffaele Hospital, Vita-Salute San Raffaele University, Milan, Italy; Experimental Imaging Center, IRCCS San Raffaele Scientific Institute, Milan, Italy; Department of Arrhythmology and Cardiac Electrophysiology, IRCCS San Raffaele Scientific Institute, Milan, Italy; Department of Arrhythmology and Cardiac Electrophysiology, IRCCS San Raffaele Scientific Institute, Milan, Italy; Experimental Imaging Center, IRCCS San Raffaele Scientific Institute, Milan, Italy; Echocardiography Laboratory, IRCCS San Raffaele Hospital, Vita-Salute San Raffaele University, Milan, Italy; Department of Cardiac Surgery, IRCCS San Raffaele Hospital, Vita-Salute San Raffaele University, Milan, Italy; Department of Cardiac Surgery, IRCCS San Raffaele Hospital, Vita-Salute San Raffaele University, Milan, Italy; Department of Cardiac Surgery, IRCCS San Raffaele Hospital, Vita-Salute San Raffaele University, Milan, Italy; Department of Arrhythmology and Cardiac Electrophysiology, IRCCS San Raffaele Scientific Institute, Milan, Italy; Department of Cardiac Surgery, IRCCS San Raffaele Hospital, Vita-Salute San Raffaele University, Milan, Italy

**Keywords:** Mitral valve repair, Mitral regurgitation, Barlow’s disease, Ventricular arrhythmias, Mitral annular disjunction

## Abstract

**OBJECTIVES:**

Aim of this study was to evaluate arrhythmic burden of patients with Barlow’s disease and significant mitral regurgitation (MR) and assess the impact of mitral repair on ventricular arrhythmias (VA) in this group of subjects.

**METHODS:**

We prospectively included 88 consecutive patients with Barlow’s disease referred to our Institution from February 2021 to May 2022. All enrolled patients underwent 24-h Holter monitoring before surgery. Sixty-three of them completed 3 months echocardiographic and Holter follow-up. Significant arrhythmic burden was defined as ≥1% premature ventricular beats/24 h or at least one episode of non-sustained ventricular tachycardia (VT), VT or ventricular fibrillation.

**RESULTS:**

At baseline, 29 patients (33%) were arrhythmogenic (AR), while 59 (67%) were not [non-arrhythmogenic (NAR)]. AR subjects tended to be more often females with history of palpitations. Sixty-three patients completed 3-months follow-up. Twenty of them (31.7%) were AR at baseline and 43 (68.3%) were not. Among AR patients, 9 (45%) remained AR after mitral surgery, while 11 (55%) became NAR. Considering NAR subjects at baseline, after mitral valve repair 8 (18.6%) evolved into AR, while 35 (81.4%) remained NAR. A higher prevalence of pre-operative MAD was found among patients experiencing VA reduction if compared with patients who remained arrhythmogenic (63.6% vs 11.1%, *P* = 0.028).

**CONCLUSIONS:**

In our experience, one-third of Barlow’s patients referred for mitral surgery showed a significant arrhythmic burden. Almost half of the subjects arrhythmogenic at baseline were free from significant VA after mitral repair. However, a minority (18.6%) of subjects without arrhythmic burden at baseline experienced significant VA at follow-up.

**Figure:**
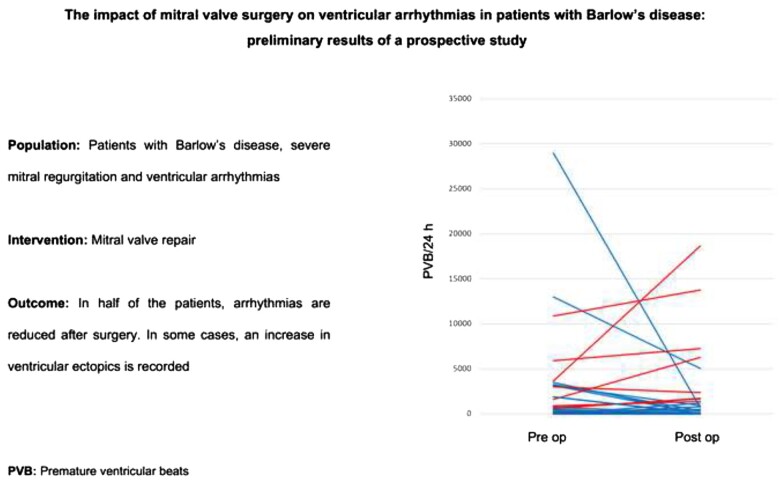

## INTRODUCTION

Mitral valve prolapse (MVP), defined as a systolic abnormal displacement of mitral leaflets above the annular plane [1], is a common finding at echocardiographic examination, with a reported prevalence around 2–3% [2].

As already noticed in Barlow’s first studies [3], a subgroup of MVP patients, namely young females with T wave inversion in infero-lateral leads at ECG [4], shows an increased risk of malignant ventricular arrhythmias (VA) and sudden cardiac death (SCD). This association has been subsequently proven by different reports [5, 6], even autoptic [7], who identified bileaflet prolapse in up to 70% of patients with MVP experiencing SCD before 40 years of age.

A recent renewed interest in this topic has fostered the identification of a specific ‘malignant arrhythmic MVP’ phenotype. Patients more prone to develop VA and SCD usually have echocardiographic characteristics of Barlow’s disease [thickened, redundant leaflets, bileaflet prolapse, elongated chordae, with or without mitral annular disjunction (MAD)] [2, 8], myocardial fibrosis at papillary muscles and infero-basal left ventricular wall (both at cardiac magnetic resonance imaging [9] and autoptic studies [6, 7]) and left ventricular contraction abnormalities (Pickelhaube sign [10] and left ventricular mechanical dispersion as assessed by speckle-tracking echocardiography [11]). Interestingly, electrophysiological studies mapped the site of VA origin in the same regions where myocardial fibrosis is usually detected [12].

Mitral regurgitation (MR) has surely a role in arrhythmogenesis in this context [13], even if the association between MVP and malignant arrhythmias has been proven even in absence of haemodynamically significant MR [6, 9].

No definitive data are available on baseline arrhythmic burden and evolution of VA in patients with Barlow’s disease undergoing mitral surgery for concomitant severe mitral regurgitation. In fact, only few case reports and retrospective series are available on the subject, with controversial results.

Therefore, we started a prospective, single-centre study with the following aims:

1. to evaluate the arrhythmic burden at baseline and to identify differences between arrhythmogenic and non-arrhythmogenic Barlow’s patients;
2. to assess the impact of mitral valve repair on ventricular arrhythmias.

## MATERIALS AND METHODS

### Ethical statement

Our institutional Ethics Committee approved this prospective study and waived individual consent from the patients (approval number 87/INT/2020).

### Study population

From February 2021 to May 2022, 650 consecutive patients were referred to our institution for surgical treatment of severe MR. Eighty-eight (13.5%) of them were affected by Barlow’s disease and were thus prospectively enrolled in this study (registration number NCT05562804).

All the patients underwent pre-operative transoesophageal echocardiography (TEE) and coronary angiogram, whenever indicated [14]. Furthermore, in a subgroup of patients, either cardiac computed tomography (CT) scan [15] or cardiac magnetic resonance imaging (MRI) [9] were performed in order to assess the presence and extent of myocardial fibrosis. Myxomatous degeneration was at first identified at pre-operative TEE and then confirmed during surgical intraoperative valve exploration. Transoesophageal echocardiography focused on MR grading and mitral valve morphology analysis, including assessment of leaflet redundancy and prolapse, chordal elongation, annular dilatation, presence of calcifications and MAD characterization [1, 16, 17].

MAD distance was measured in the parasternal long-axis view at end-systole, from the insertion of the posterior leaflet on the detached mitral annulus to the border of the bulging left ventricle [18, 19].

All enrolled patients underwent a pre-operative 24-h Holter recording. ECG traces were analysed in terms of heart rhythm and heart rate. Presence and burden of premature ventricular beats (PVB) over 24 h were assessed. PVB morphology was classified as papillary muscle morphology, annular morphology or other sites, according with Al’Aref *et al.* [20]. Non-sustained ventricular tachycardia (NSVT) and sustained ventricular tachycardia (VT) occurrence was also noted. Significant arrhythmic burden was defined as ≥1% PVB/24 h or at least one episode of NSVT, VT or ventricular fibrillation (VF) [9]. According to these criteria, patients were classified in arrhythmogenic and non-arrhythmogenic. All patients underwent transthoracic echocardiography right before hospital discharge. The pre-operative, intraoperative and post-operative data were prospectively entered in a dedicated database and compared between arrhythmogenic and non-arrhythmogenic subjects.

### Follow-up

All enrolled patients were involved in a follow-up protocol, with the first reevaluation at 3 months, including 24-h Holter monitoring and transthoracic echocardiography.

Data were collected from our institutional outpatient clinic visits, or by means of telephone interviews with the patients and the referring cardiologists; we focused on survival, cardiac reoperation, changes in arrhythmic burden, current therapy and symptoms.

Antiarrhythmic therapy was defined as increased when patients not on antiarrhythmic drugs at baseline received a new antiarrhythmic medication at follow-up, or if a shift from low dosage beta-blockers to high dosage beta-blockers or class Ic/III drugs occurred.

### Statistical analyses

Categorical data were described as absolute and percentage (%) frequency values and compared with the χ^2^ or the Fisher exact tests, as appropriate. The Shapiro–Wilk test was used to assess whether the distribution was normal or not-normal. Continuous normally distributed variables were expressed as mean ± standard deviation (SD) and compared with paired *t*-test or *t*-test for independent samples. Continuous not-normal variables were reported as median (25th percentile; 75th percentile) and compared with Wilcoxon signed-rank test for related samples or with Mann–Whitney test for unrelated samples.

Patients arrhythmogenic or not at follow-up in each group (i.e. with or without significant arrhythmias at baseline) were represented by absolute and percentage frequency values.

Incidence rates along with 95% confidence intervals were calculated at follow-up for each outcome (i.e. presence/absence of significant arrhythmic burden, at least one episode of NSVT/VT/VF, PVB>1%) per 100 person-years at risk.

A *P*-value of <0.05 was used to define statistical significance.

## RESULTS

The preoperative clinical characteristics of the study population are shown in Table 1. Among 88 enrolled patients [median age 55 years (44–63)], 36 (40.9%) were female. Twenty-six subjects (31.3%) had family history of Barlow’s disease. Eleven (12.6%) were affected by pre-operative atrial fibrillation (AF). Median left ventricular ejection fraction (LVEF) was 61% (58–65) and median left ventricular end diastolic diameter was 52.5 mm (48–57.25). Median mitral valve area was 7.3 cm^2^ (6.1–9). No patients had history of myocardial infarction, hypertrophic cardiomyopathy or significant aortic stenosis with left ventricular hypertrophy.

**Table 1:** Pre-operative patients’ characteristics (88 patients)

	Overall (88 patients)	Group 1 Arrhythmogenic (29 patients)	Group 2 Non-arrhythmogenic (59 patients)	*P*-value
Female sex, *n* (%)	36 (40.9)	16 (55.2)	20 (33.9)	0.056
Age (years), median (IQR)	55 (44–63)	56.5 (45.0–71.5)	53 (44–61)	0.174
Family history of Barlow’s disease, *n* (%)	26 (31.3)	7 (25.9)	19 (33.9)	0.461
Atrial fibrillation, *n* (%)	11 (12.6)	3 (10.7)	8 (13.6)	0.709
Hypertension, *n* (%)	23 (26.4)	10 (35.7)	13 (22.0)	0.176
Smoke, *n* (%)	31 (35.6)	9 (31.1)	22 (37.3)	0.640
Tricuspid valve regurgitation > 2, *n* (%)	6 (6.8)	2 (6.9)	4 (6.8)	0.999
LVEF (%), median (IQR)	61 (58–65)	60 (58–65)	61 (58–65)	0.863
LV-EDD (mm), median (IQR)	52.5 (48– 57.25)	52 (46.2–54.7)	54 (48–58)	0.490
Left atrial volume (ml), median (IQR)	76 (59.75– 99.25)	72 (59–103)	76 (62.5–96.5)	0.881
sPAP (mmHg), median (IQR)	30 (25–38)	31 (27–42)	30 (25–35)	0.137
Mitral valve area (cm^2^), median (IQR)	7.3 (6.1–9)	8 (6–10)	7 (6.3–8.5)	0.670
A-P mitral annulus (mm), median (IQR)	34 (30–38)	35 (31–38)	34 (30–38)	0.872
IC mitral annulus (mm), median (IQR)	43 (41–47.25)	43.5 (41–48)	43 (40–47)	0.687
Bileaflet prolapse, *n* (%)	59 (67.8)	20 (71.4)	30 (66.1)	0.619
Prevalent anterior leaflet prolapse, *n* (%)	3 (3.4)	1 (3.6)	2 (3.4)	0.999
Prevalent posterior leaflet prolapse, *n* (%)	25 (28.7)	7 (25.0)	18 (30.5)	0.800
Infero-lateral MAD, *n* (%)	30 (34.5)	11 (39.3)	19 (32.2)	0.516
Infero-lateral MAD length (mm), median (IQR)	8.5 (6–10)	9 (6.5–11.5)	8 (6–9.5)	0.337
Pickelhaube sign, *n* (%)	27 (31.0)	12 (42.9)	15 (25.4)	0.101
Myocardial fibrosis, *n* (%)	4 (4.5)	2 (6.9)	2 (3.4)	0.458
Annular calcifications, *n* (%)	14 (16.1)	7 (25.0)	7 (11.9)	0.119
PVB/24 h, median (IQR)	171.5 (8–800.5)	2000 (728.5–4730)	24 (4–198)	<0.001
PVB/24 h ≥5%, *n* (%)	7 (7.9)	7 (25.0)	0	–
NSVT, *n* (%)	19 (21.6%)	19 (65.5)	0	–
VT, *n* (%)	1 (1.1)	1 (3.4)	0	–
VF, *n* (%)	3 (3.4)	3 (10.3)	0	–
NTW in inferior leads, *n* (%)	22 (32.4)	10 (47.6)	12 (25.5)	0.720
Polymorphic PVB, *n* (%)	56 (67.5)	24 (88.9)	32 (57.1)	0.004
PVB morphology, *n* (%)				
PMP	26 (41.9)	11 (68.8)	15 (32.6)	0.012
ALP	2 (3.2)	1 (6.3)	1 (2.2)	0.453
AN	14 (22.6)	7 (43.8)	7 (15.2)	0.019
RVOT	18 (20.4)	10 (34.5)	8 (13.5)	0.045
Other sites	26 (29.5)	12 (41.4)	14 (23.7)	0.145
Significant PVB morphology, *n* (%)	35 (56.5)	14 (87.5)	21 (45.7)	0.004
Palpitations, *n* (%)	44 (50.6)	20 (71.4)	24 (40.7)	0.007
Syncope, *n* (%)	9 (10.3)	5 (17.9)	4 (6.8)	0.140
NYHA class, *n* (%)				0.966
I	23 (26.2)	8 (27.6)	15 (25.4)	
II	65 (73.8)	21 (72.4)	44 (74.6)	
Antiarrhythmic therapy, *n* (%)	34 (40.0)	14 (53.8)	20 (33.9)	0.084
Class Ic	3 (3.5)	1 (3.8)	2 (3.4)	0.999
Class II	31 (36.5)	11 (42.3)	20 (33.9)	0.458
Class III	3 (3.5)	2 (7.7)	1 (1.7)	0.220

ALP: antero-lateral papillary muscle; AN: annular; A-P: anterior-posterior; IC: intercommissural; IQR: interquartile range; LV-EDD: left ventricular end-diastolic diameter; LVEF: left ventricular ejection fraction; MAD: mitral annular disjunction; NSVT: non-sustained ventricular tachycardia; NTW: negative T waves; NYHA: New York Heart Association; PMP: postero-medial papillary muscle; PVB: premature ventricular beats; RVOT: right ventricular outflow tract; sPAP: systolic pulmonary artery pressure; VT: ventricular tachycardia; VF: ventricular fibrillation.

Fifty-nine patients (67.8%) were affected by bileaflet mitral valve prolapse, while in 25 cases (28.7%) a prevalent prolapse of the posterior leaflet was identified. Thirty subjects (34.5%) had documented MAD, and it was infero-lateral in all the cases. Twenty-seven patients (31%) showed positive Pickelhaube sign at tissue Doppler imaging. The median MAD length was 8.5 mm (6–10). Fourteen subjects (16.1%) had mitral annular calcifications.

Sixteen patients (18.2%) underwent a pre-operative cardiac CT scan/MRI. In four of them, myocardial fibrosis was detected and was located at the level of the infero-basal left ventricular wall, near mitral valve annulus.

Overall, the median number of PVB/24 h identified at pre-operative Holter monitoring was 171.5 (8–800.5). Fifty-six patients (67.5%) had polymorphic PVB. Twenty-eight subjects (45.1%) showed PVB with papillary muscles morphology, while in 14 cases (22.6%), an annular PVB morphology was identified. Nineteen patients (21.6%) had documented NSVT episodes. Four patients (4.5%) had an implantable cardioverter-defibrillator (ICD) as a secondary prevention tool (three patients with history of VF and one with history of symptomatic VT). Forty-four patients (50.6%) complained of palpitations. All the enrolled subjects were in New York Heart Association (NYHA) functional class I or II. At the time of pre-operative Holter, 34 patients (40%) were on antiarrhythmic therapy. Details on antiarrhythmic drugs are shown in Table 1.

### Operative details

Operative details are shown in Table 2. Eighty-one patients (92%) underwent mitral valve repair, while five patients had mitral valve replacement and other two transcatheter mitral valve repair and were thus excluded from further analysis (Fig. 2).

**Table 2:** Operative details and post-operative outcomes (88 patients)

	Overall (88 patients)	Group 1 Arrhythmogenic (29 patients)	Group 2 Non-arrhythmogenic (59 patients)	*P*-value
Mitral valve repair technique, *n* (%)				
Posterior leaflet resection	14 (16.1)	5 (17.9)	9 (15.3)	0.762
Central edge-to-edge	56 (64.4)	19 (67.9)	37 (62.7)	0.811
Edge-to-edge A1-P1	3 (3.4)	1 (3.6)	2 (3.4)	0.999
Edge-to-edge A3-P3	3 (3.4)	0 (0.0)	3 (5.1)	–
Neochordae, posterior leaflet	11 (12.6)	2 (7.1)	9 (15.3)	0.491
Neochordae, anterior leaflet	4 (4.6)	0	4 (6.8)	–
Prosthetic ring size (mm), median (IQR)	38 (37–39)	38 (37–39)	38 (37–39)	0.882
Mitral valve replacement, *n* (%)	5 (5.7)	2 (7.1)	3 (5.1)	0.999
TEER, *n* (%)	2 (2.3)	1 (3.6)	1 (1.7)	0.999
Concomitant procedures, *n* (%)	29 (33.3)	10 (35.7)	19 (32.2)	0.810
Tricuspid valve repair, *n* (%)	15 (17)	6 (20.7)	9 (15.2)	0.524
AF ablation, *n* (%)	8 (9)	1 (3.4)	7 (11.9)	0.197
Aortic valve replacement, *n* (%)	2 (2.3)	1 (3.4)	1 (1.7)	0.603
CABG, *n* (%)	4 (4.5)	1 (3.4)	3 (5)	0.729
Re-exploration, *n* (%)	2 (2.3)	1 (3.6)	1 (1.7)	0.543
LCOS, *n* (%)	2 (2.3)	1 (3.6)	1 (1.7)	0.543
Post-operative AF, *n* (%)	24 (27.6)	10 (35.7)	14 (23.7)	0.306
Aortic cross clamp time (min), median (IQR)	68 (52–83.7)	58 (50.2–91.7)	71 (53.2–83)	0.437
CPB time (min), median (IQR)	89 (70–113)	84.5 (70–113)	90 (69.5–114.5)	0.633
Second pump run, *n* (%)	0	0 (0.0)	3 (5.1)	–
Hospital LOS (days), median (IQR)	5 (4–7)	6 (5–7)	5 (4–7)	0.231
LVEF at discharge (%), median (IQR)	56 (55–60)	57 (55–60)	55 (55–60)	0.940

AF: atrial fibrillation; CABG: coronary artery bypass grafting; CPB: cardiopulmonary bypass; IQR: interquartile range; LCOS: low cardiac output syndrome; LOS: length of stay; LVEF: left ventricular ejection fraction; TEER: transcatheter edge to edge repair

Most of the patients (56, 64.4%) underwent A2-P2 edge to edge procedure, while in 14 subjects (16.1%), the technique of choice was quadrangular resection of the posterior leaflet. In the mitral valve repair group, leaflet manipulation was always followed by an annuloplasty with a posterior band [median ring size 38 mm (37–39)].

Twenty-nine patients (33.3%) underwent concomitant procedures. In particular, tricuspid valve repair was performed in 15 subjects (17%), 2 patients (2.3%) underwent aortic valve replacement for coexistent severe aortic regurgitation and in 4 cases (4.7%) a concomitant coronary artery bypass grafting was performed. However, no one of these patients had history of angina or previous myocardial infarction, nor ventricular segmental wall motion abnormalities at pre-operative echocardiography. In all the cases, a 70% coronary stenosis was occasionally discovered during pre-operative coronary angiography and subsequently treated.

### In-hospital outcomes

**Figure 1:**
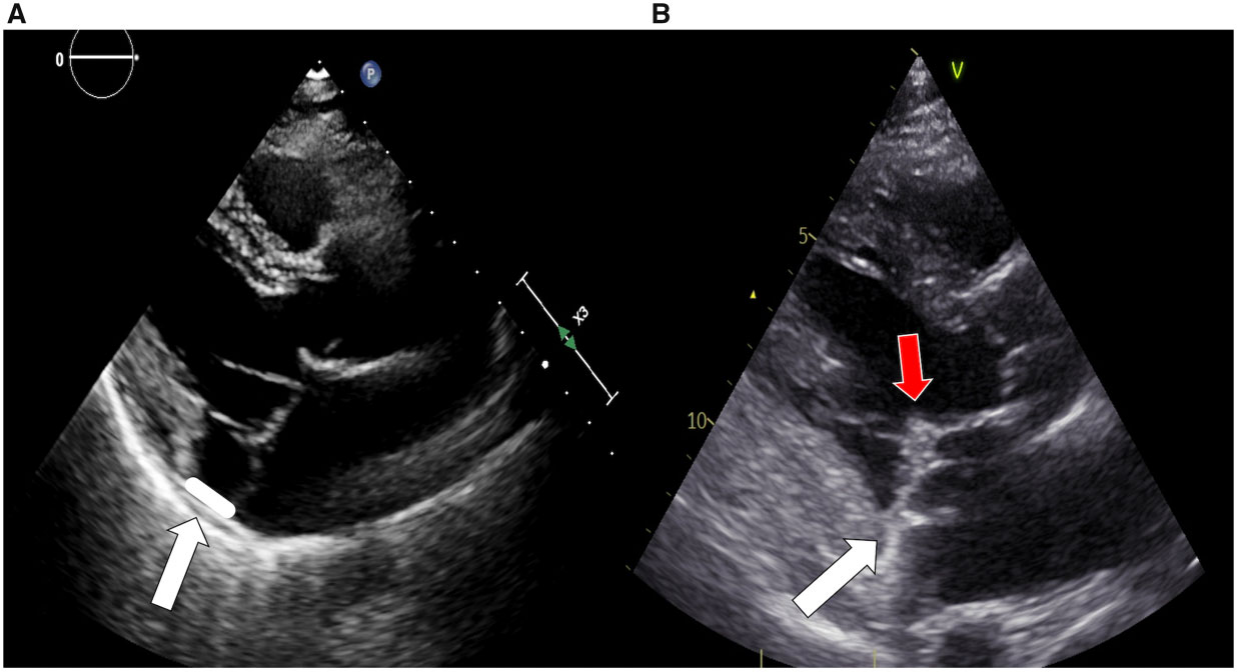
Parasternal long-axis view at transthoracic echocardiography of pre-operative and post-operative mitral valve. White arrows point at atrioventricular junction: pre-operative MAD (**A**) and its post-operative disappearance (**B**). Red arrow indicates the coaptation point, clearly below the annular plane.

There were no in-hospital death. The occurrence of the most common post-operative complications is shown in Table 2. Two patients (2.3%) required re-exploration for post-operative bleeding. Post-operative AF was experienced by 24 subjects (27.6%). The median post-operative in hospital length of stay was 5 days (4–7). At discharge, all patients had no or mild MR and were free from MAD (Fig. 1).

### Comparison between arrhythmogenic and non-arrhythmogenic patients

At baseline, 29 patients (33%) were classified as arrhythmogenic (group 1), while 59 (67%) were non-arrhythmogenic (group 2). The median PVB number/24 h was 2000 (728.5–4730) in group 1 and 24 (4–198) in group 2 (*P* < 0.001). Group 1 patients were more frequently affected by polymorphic PVB [24 subjects (88.9%) vs 32 (57.1%) in group 2, *P* = 0.004]. Both papillary muscles and annular PVB morphology were more common in Group 1 (Table 1).

Arrhythmogenic patients tended to be more often females (55.2% in group 1 vs 33.9% in group 2, *P* = 0.056) with history of palpitations [20 group 1 patients (71.4%) vs 24 group 2 patients (40.7%), *P* = 0.007]; no other significant differences were found in the pre-operative, intraoperative and post-operative characteristics. Interestingly, infero-lateral MAD prevalence was similar between groups [11 group 1 patients (39.3%) vs 19 group 2 patients (32.2%), *P* = 0.516].

### Follow-up outcomes

Sixty-three patients who underwent mitral valve repair had at least one follow-up at 3 months, while the remaining did not reach yet the scheduled re-evaluation time point and were thus excluded from further analysis. Patients’ characteristics at follow-up are shown in Table 3. There were no deaths nor REDO cases. All but two patients showed no or mild MR. Median EF was 60% (55–60).

**Table 3:** Follow-up outcomes (63 patients who underwent mitral valve repair)

Mitral valve regurgitation, *n* (%)	
0	42 (66.7)
1	19 (30.1)
3	2 (3.2)
LVEF (%), median (IQR)	55 (55–60)
Antiarrhythmic therapy, *n* (%)	54 (85.7)
Ic	1 (1.6)
II	53 (84.1)
III	3 (4.8)
Increase in antiarrhythmic therapy dosage, *n* (%)	10 (15.9)
PVB/24 h, median (IQR)	26 (6.5–272.7)
PVB/24 h ≥5%, *n* (%)	5 (7.9)
NSVT, *n* (%)	11 (17.5)
AF, *n* (%)	2 (3.2)
NYHA class, *n* (%)	
I	7 (11.1)
II	56 (88.9)

AF: atrial fibrillation; LVEF: left ventricular ejection fraction; PVB: premature ventricular beats; NSVT: non-sustained ventricular tachycardia; NYHA: New York Heart Association.

Fifty-four patients (85.7%) were on antiarrhythmic medications. Of them, 10 patients (15.9%) received an increased antiarrhythmic therapy dosage if compared with baseline. All but three patients were NYHA functional class I or II. Two patients (3.2%) experienced AF during follow-up. Both had history of pre-operative paroxysmal AF, and no changes in antiarrhythmic medications were registered in any of the cases.

**Figure 2:**
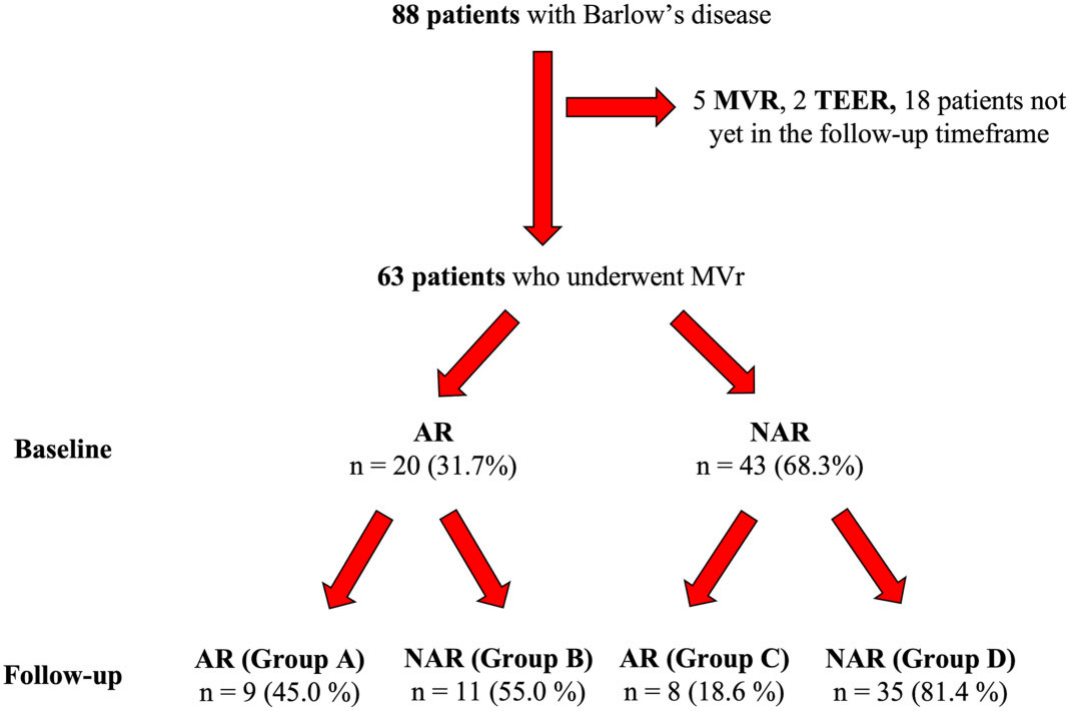
Flow-chart showing arrhythmic burden evolution of enrolled patients. AR: arrhythmogenic patients; MVR: mitral valve replacement; MVr: mitral valve repair; NAR: non-arrhythmogenic patients; TEER: transcatheter edge to edge repair.

**Figure 3:**
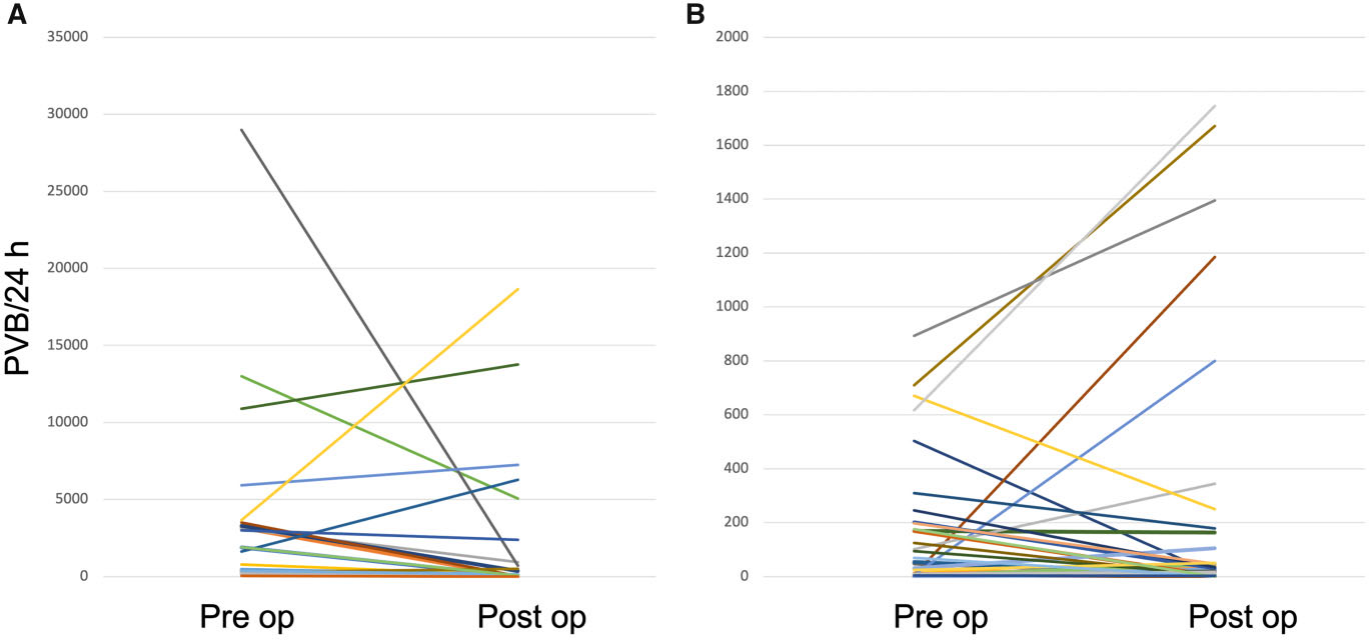
PVB trend at follow-up in patients who were arrhythmogenic (**A**) and non-arrhythmogenic (**B**) at baseline.

Out of 63 patients who completed 3 months follow-up, twenty of them (31.7%) were arrhythmogenic at baseline and 43 (68.3%) were not. Among arrhythmogenic patients, 9 (45%) remained arrhythmogenic after mitral surgery (group A), while 11 (55%) showed no significant arrhythmic burden at follow-up (group B). Among non-arrhythmogenic subjects, a significant arrhythmic burden appeared after mitral valve repair in 8 patients (18.6%, Group C), while 35 (81.4%) remained non-arrhythmogenic (group D). Changes in the arrhythmic burden are shown in Figures 2 and 3 and in Central Image and a comparison between groups is shown in Supplementary Table S1. Interestingly, among patients arrhythmogenic at baseline, infero-lateral MAD was found significantly more often in group B than group A patients (63.6% vs 11.1%, *P* = 0.028), while no differences were found in intraoperative and in-hospital outcomes (including surgical technique). The incidence rate of absence of significant VA at follow-up among patients arrhythmogenic at baseline was 55 (27.5–98.4) per 100 person-years at risk. On the other hand, the incidence rate of significant arrhythmic burden at follow-up among non-arrhythmogenic patients was 18.6 (8–36.7) per 100 person-years at risk. Specifically, the incidence of NSVT, VT or VF was 14 (5.1–30.4) per 100 person-years at risk, while the incidence of PVB >1%/24 h was 9.3 (2.5–23.8) per 100 person-years at risk.

## DISCUSSION

To the best of our knowledge, the present study represents the first prospective analysis of VA burden in Barlow’s patients undergoing mitral valve surgery. Data showed that, in this population, one-third of subjects have a significant arrhythmic burden before surgery, which is not uniformly affected by mitral repair. In fact, almost half of the patients with significant VA at baseline were free from them after mitral repair, while a minority (18.6%) of subjects without arrhythmic burden at baseline developed a significant arrhythmic burden during follow-up.

### Comparison between arrhythmogenic and non-arrhythmogenic patients

Several retrospective series described echocardiographic and arrhythmic characteristics of MVP patients. The reported median number of PVB/24 h varies considerably between different studies, ranging from 41 (16–196) [21] to almost 4000 (0–3000) [22]. In our report, the median number of PVB/24 h at baseline was 171.5 (8–800.5). We found that arrhythmogenic patients tended to be females with history of palpitations. This is consistent with the ‘malignant arrhythmic MVP’ phenotype already described elsewhere [4, 8]. Almost 35% of patients in our population had MAD. Similarly, Essayagh *et al*. [23] reported a MAD prevalence of 44% among 61 patients with MVP and severe MR. In our cohort, MAD was infero-lateral in all the cases. This is consistent with Zugwitz *et al*. [18], who recently proved that disjunction at any location is quite common in the general population (up to 76% of cases), while its infero-lateral occurrence is rare and associated with MVP. We found no difference in MAD prevalence between arrhythmogenic and non-arrhythmogenic patients. Even if the association between MAD and VA has been recently established in large retrospective series [8, 9, 22], the anatomical substrate of annular disjunction is still unclear and a proper distinction between ‘true’ MAD and ‘pseudo’ MAD may be difficult with echocardiographic imaging, thus leading to a global overestimation of MAD prevalence in MVP patients [24]. Further studies are needed to better characterize mitral annular disjunction and its association with arrhythmic events.

### Effects of mitral valve repair on ventricular arrhythmias

The role of mitral valve surgery on ventricular arrhythmias in patients with Barlow’s disease is still object of debate. In the arrhythmogenesis of these patients, several triggers have been proposed as electrophysiological substrate, namely MR itself [13], prolapse-induced myocardial fibrosis [9] and MAD [22]. Mitral valve repair, through mitral leaflets prolapse correction, MR abolition and annular stabilization, may theoretically mitigate the causes of electrical instability, but only few case reports [25, 26] and small case series [27, 28] are available on the subject, with controversial results.

In our study, mitral valve repair did not uniformly affect VA burden in patients with Barlow’s disease. About half of patients with an arrhythmogenic profile at baseline were free from significant VA after surgery, while less than 20% of those without arrhythmic burden at baseline developed significant VA during follow-up. The potential ineffectiveness of surgery on VA in this context has already been reported. Naksuk et al. [21] depicted no changes in overall arrhythmic burden in 32 patients with bileaflet mitral valve prolapse and severe MR undergoing mitral valve surgery. However, a slight reduction of PVB frequency (>10%) was recorded in younger patients. In our experience, no differences were found in terms of age between patients experiencing VA reduction and patients who did not (Supplementary Material, Table S1). On the other hand, Essayagh *et al*. [22] showed in a large retrospective cohort of 595 patients the ability of mitral valve surgery to reduce MAD-related excess of arrhythmic events, when compared with medical management. However, no information is available on surgical techniques, and 7% of their patients underwent mitral valve replacement.

In our experience, independently from the repair technique, leaflet coaptation moved towards the apex, and below the annular plane, thus abolishing the prolapse-triggered stretch on papillary muscles (Fig. 1). However, most of our population underwent edge to edge mitral repair, thus a proper comparison between different techniques was not feasible.

In 18.6% of non-arrhythmogenic patients of our cohort, successful repair (with complete abolition of MAD) was followed by an increase in VA at 3-month follow-up. This finding indicates the possibility of a myocardial excitability progression during Barlow’s subjects natural history, irrespective of mitral surgery. This is consistent with Brunec-Keller *et al.* [29] results, suggesting pre-operative bileaflet MVP as an independent risk factor for increased VA burden after surgery. In our cohort, however, no differences in bileaflet MVP prevalence were recorded between groups.

Moreover, we found a higher prevalence of pre-operative infero-lateral MAD among patients experiencing VA reduction, compared with patients who remained arrhythmogenic. Considering that a complete abolition of MAD was achieved in all the subjects through surgical annuloplasty (Fig. 1), the existence of this subgroup suggests that in patients without MAD another electric trigger may exist, not addressed by mitral valve repair.

Further studies on larger samples are needed to confirm these findings and identify predictors of VA decrease/increase after surgery. A risk stratification protocol to clearly establish who may benefit from loop recorder or even ICD implantation, especially in the group who experienced an increase in arrhythmic burden after surgery, is also mandatory [19].

### Limitations

The main limitation of this study is the small sample size, who limited statistical analysis (i.e. the identification of predictors of VA decrease/increase after surgery). Second, only 18.2% of our population underwent a proper tissue characterization with either MRI or CT scan, thus we were not able to report on arrhythmia and fibrosis. Third, only data from one follow-up window were available; therefore, a continuous evaluation of VA evolution after surgery was not yet feasible. Our protocol is ongoing to overcome those limitations in the next future.

## CONCLUSIONS

In our experience, one-third of Barlow’s patients with severe MR, referred for mitral surgery, showed a significant arrhythmic burden. These patients more frequently experienced palpitations and were more commonly affected by polymorphic PVB if compared with non-arrhythmogenic patients, but no other differences were found in clinical or echocardiographic characteristics (including MAD prevalence) between these two groups.

Almost half of the patients with arrhythmic burden at baseline were free from significant VA after mitral repair. However, a minority of subjects without arrhythmic burden at baseline experienced a significant PVB burden at follow-up.

Our prospective study will continue to enrol patients to better define the role of MV repair on VA in a larger cohort of patients, with a longer follow-up.

## Supplementary Material

ivad073_Supplementary_Data

## Data Availability

The data underlying this article will be shared on reasonable request to the corresponding author.

## Notes

Presented at the 36th EACTS Annual Meeting, Milan, Italy, 5–8 October 2022.
