# Supplementary material for: The impact of mitral valve surgery on ventricular arrhythmias in patients with Barlow’s disease: preliminary results of a prospective study
Source: Interdiscip Cardiovasc Thorac Surg. 2023 May 11;36(5):ivad073. doi: 10.1093/icvts/ivad073 (PMC10212537; doi:10.1093/icvts/ivad073)
Supplement: ivad073_Supplementary_Data [file ivad073_supplementary_data.docx]

**Supplementary Table 1.** Comparison between patients with different arrhythmic burden evolutions.

|  | **Group A**  **(9 patients)** | **Group B**  **(11 patients)** | **Group C**  **(8 patients)** | **Group D**  **(35 patients)** |  |  |
| --- | --- | --- | --- | --- | --- | --- |
| **Pre-operative characteristics** |  |  |  |  |  |  |
| **Female sex** (n, %) | 6 (66.7) | 3 (27.3) | 3 (37.5) | 14 (40) |  |  |
| **Age, years** (median, IQR) | 55 [48.5; 69] | 51 [40; 63] | 59.5 [46.5; 64.2] | 52 [41; 59] |  |  |
| **Tricuspid valve regurgitation > 2** (n, %) | 0 (0) | 1 (9.1) | 1 (12.5) | 1 (2.9) |  |  |
| **LVEF, %** (median, IQR) | 58 [56; 60] | 62 [58; 65] | 61 [57; 65] | 62 [58; 65] |  |  |
| **LV-EDD, mm** (median, IQR) | 52 [45; 54.5] | 54 [52; 59] | 54 [49; 57] | 51 [46; 58] |  |  |
| **sPAP, mmHg** (median, IQR) | 35 [27; 46.5] | 30 [22.7; 38.2] | 30 [25; 45] | 29 [25; 32.2] |  |  |
| **Bileaflet prolapse** (n, %) | 6 (66.7) | 7 (63.6) | 7 (87.5) | 23 (65.7) |  |  |
| **Infero-lateral MAD** (n, %) | 1 (11.1) | 7 (63.6) | 2 (25) | 16 (45.7) |  |  |
| **Infero-lateral MAD length, mm** (median, IQR) | 10 [10; 10] | 7 [6; 11] | 8.5 [8.25; 8.75] | 5.75 [7; 10] |  |  |
| **Pickelhaube sign** (n, %) | 1 (11.1) | 6 (54.5) | 3 (37.5) | 11 (31.4) |  |  |
| **PVB/24 hours** (median, IQR) | 3101 [996; 8397.5] | 1889 [266; 3277] | 463 [29.2; 699.2] | 8 [2; 94] |  |  |
| **PVB/24 hours ≥ 5%** (n, %) | 3 (33.3) | 1 (9) | 0 | 0 |  |  |
| **NSVT** (n, %) | 7 (77.8) | 7 (63.6) | 0 | 0 |  |  |
| **VT** (n, %) | 0 | 0 | 0 | 0 |  |  |
| **VF** (n, %) | 0 | 2 (18.2) | 0 | 0 |  |  |
| **NTW in inferior leads** (n, %) | 5 (55.5) | 5 (45.4) | 1 (25) | 8 (26.7) |  |  |
| **Polymorphic PVB** (n, %) | 8 (88.9) | 8 (80.0) | 5 (62.5) | 15 (42.9) |  |  |
| **PVB morphology:** | |  |  |  |  | |
| - **PMP** (n, %) | 6 (85.7) | 2 (50) | 1 (25) | 10 (34.5) |  |  |
| - **ALP** (n, %) | 0 (0) | 0 (0) | 0 | 1 (2.9) |  |  |
| - **AN** (n, %) | 2 (28.6) | 2 (50) | 1 (25) | 1 (3.4) |  |  |
| - **RVOT** (n, %) | | 4 (44.4) | 3 (27.3) | 2 (25.0) | 4 (11.4) | |
| - **Other sites** (n, %) | | 2 (22.2) | 4 (36.4) | 2 (25.0) | 4 (11.4) | |
| **Significant PVB morphology** (n, %) | | 6 (85.7) | 3 (75) | 1 (25) | 12 (41) | |
| **Palpitations** (n, %) | 8 (88.9) | 7 (63.6) | 4 (50) | 16 (45.7) |  |  |
| **Antiarrhythmic therapy** (n, %) | 2 (25.0) | 5 (55.6) | 4 (50) | 10 (28.6) |  |  |
| - **Class Ic** | 0 | 0 | 1 (12.5) | 1 (2.9) |  |  |
| - **Class II** | 1 (11.1) | 5 (55.6) | 4 (50) | 10 (28.6) |  |  |
| - **Class III** | 1 (11.1) | 0 | 1 (12.5) | 0 |  |  |
| **Intra-operative and post-operative outcomes** |  |  |  |  |  |  |
| **Surgical technique:** |  |  |  |  | |  |
| - **Posterior leaflet resection** (n, %) | 1 (11.1) | 3 (27.3) | 2 (25) | 6 (17.1) | |  |
| - **Central edge-to-edge** (n, %) | 7 (77.8) | 7 (63.6) | 6 (75) | 24 (68.6) | |  |
| - **Edge-to-edge A1-P1** (n, %) | 1 (11.1) | 0 | 0 | 2 (5.7) | |  |
| - **Edge-to-edge A3-P3** (n, %) | 0 | 0 | 0 | 3 (8.6) | |  |
| - **Neochordae, posterior leaflet** (n, %) | 1 (11.1) | 1 (9.1) | 1 (12.5) | 4 (11.4) | |  |
| - **Neochordae, anterior leaflet** (n, %) | 0 | 0 | 0 | 1 (2.9) | |  |
| **Prosthetic ring size, mm** (median, IQR) | 39.0 [37; 39.5] | 39 [37; 39] | 37.5 [35.2; 39] | 38 [37; 39] | |  |
| **Concomitant procedures** (n, %) | 5 (55.6) | 4 (36.4) | 4 (50) | 10 (28.6) | |  |
| **LVEF at discharge, %** (median, IQR) | 55 [52.5; 60] | 58 [50; 60] | 55 [55; 60] | 60 [55; 60] | |  |
| **Follow-up outcomes** |  |  |  |  |  |  |
| **Mitral valve regurgitation** (n, %) | |  |  |  |  |  |
| - **0** | | 6 (66.7) | 5 (45.5) | 3 (37.5) | 28 (84.8) |  |
| - **1** | | 1 (11.1) | 5 (45.5) | 4 (50) | 5 (15.2) |  |
| - **3** | | 0 | 1 (9.1) | 1 (12.5) | 0 |  |
| **LVEF, %** (median, IQR) | | 55 [52; 60] | 55 [52; 56] | 60 [57; 60.7] | 60 [55; 60] |  |
| **AR therapy** (n, %) | | 6 (66.7) | 8 (72.7) | 6 (75.0) | 34 (97.1) |  |
| - **Class Ic** | | 0 | 0 | 0 | 1 (2.9) |  |
| - **Class II** | | 6 (66.7) | 8 (72.7) | 6 (75) | 33 (94.3) |  |
| - **Class III** | | 0 | 1 (9.1) | 0 | 2 (5.7) |  |
| **Increase in AR therapy dosage** (n, %) | | 1 (11.1) | 2 (18.2) | 0 | 7 (20) |  |
| **PVB/24 hours** (median, IQR) | | 5661 [329; 12143.5] | 90 [20; 351] | 765 [195.7; 1602] | 10 [2; 31] |  |
| **PVB/24 hours ≥ 5%** (n, %) | 5 (55.5) | 0 | 0 | 0 |  |  |
| **NSVT** (n, %) | | 5 (25) | 0 | 6 (14) | 0 |  |

AR: antiarrhythmic; ALP: antero-lateral papillary muscle; AN: annular; IQR: interquartile range; LV-EDD: left ventricular end-diastolic diameter; LVEF: left ventricular ejection fraction; MAD: mitral annular disjunction; NSVT: non-sustained ventricular tachycardia; NTW: negative T waves; PMP: postero-medial papillary muscle; PVB: premature ventricular beats; RVOT: right ventricular outflow tract; sPAP: systolic pulmonary artery pressure.

**Group A:** patients arrhythmogenic at baseline and still arrhythmogenic after mitral valve repair

**Group B:** patients arrhythmogenic at baseline but free from significant ventricular arrhythmias at follow-up

**Group C:** patients non-arrhythmogenic at baseline but arrhythmogenic at follow-up

**Group D:** patients non-arrhythmogenic at baseline who remained free from significant ventricular arrhythmias after mitral valve repair
